# A comparative study on the effects of systemic manual acupuncture, periauricular electroacupuncture, and digital electroacupuncture to treat tinnitus: A randomized, paralleled, open-labeled exploratory trial

**DOI:** 10.1186/s12906-017-1589-3

**Published:** 2017-01-31

**Authors:** Bong Hyun Kim, Kyuseok Kim, Hae Jeong Nam

**Affiliations:** 1 Department of Clinical Korean Medicine, Graduate school Kyung Hee University, 26 Kyungheedae-ro, Dongdaemun-gu, Seoul 130-701 Republic of Korea; 2 Department of Ophthalmology, Otorhinolaryngology of Korean Medicine, College of Korean Medicine, Kyung Hee University, 26, Kyungheedae-ro, Dongdaemun-gu, Seoul 130-701 Republic of Korea

**Keywords:** Tinnitus, Systemic manual acupuncture, Electroacupuncture, Periauricular acupoints, Distal acupoints

## Abstract

**Background:**

Many previous studies of electroacupuncture used combined therapy of electroacupuncture and systemic manual acupuncture, so it was uncertain which treatment was effective. This study evaluated and compared the effects of systemic manual acupuncture, periauricular electroacupuncture and distal electroacupuncture for treating patients with tinnitus.

**Methods:**

A randomized, parallel, open-labeled exploratory trial was conducted. Subjects aged 20–75 years who had suffered from idiopathic tinnitus for > 2 weeks were recruited from May 2013 to April 2014. The subjects were divided into three groups by systemic manual acupuncture group (MA), periauricular electroacupuncture group (PE), and distal electroacupuncture group (DE). The groups were selected by random drawing. Nine acupoints (TE 17, TE21, SI19, GB2, GB8, ST36, ST37, TE3 and TE9), two periauricular acupoints (TE17 and TE21), and four distal acupoints (TE3, TE9, ST36, and ST37) were selected. The treatment sessions were performed twice weekly for a total of eight sessions over 4 weeks. Outcomes were the tinnitus handicap inventory (THI) score and the loud and uncomfortable visual analogue scales (VAS). Demographic and clinical characteristics of all participants were compared between the groups upon admission using one-way analysis of variance (ANOVA). One-way ANOVA was used to evaluate the THI, VAS _loud_, and VAS _uncomfortable_ scores. The least significant difference test was used as a post-hoc test.

**Results:**

Thirty-nine subjects were eligible and their data were analyzed. No difference in THI and VAS _loudness_ scores was observed in between groups. The VAS _uncomfortable_ scores decreased significantly in MA and DE compared with those in PE.

Within the group, all three treatments showed some effect on THI, VAS _loudness_ scores and VAS _uncomfortable_ scores after treatment except DE in THI.

**Conclusions:**

There was no statistically significant difference between systemic manual acupuncture, periauricular electroacupuncture and distal electroacupuncture in tinnitus. However, all three treatments had some effect on tinnitus within the group before and after treatment. Systemic manual acupuncture and distal electroacupuncture have some effect on VAS _uncomfortable_.

**Trial registration:**

KCT0001991 by CRIS (Clinical Research Information Service), 2016-8-1, retrospectively registered.

## Background

Tinnitus is the perception of noise in the absence of an acoustic stimulus. The number of patients with tinnitus is increasing and various factors, including stress, interference due to social interactions, noise and an aging society are associated with the increase [1, 2].

Acupuncture is commonly used to treat tinnitus in East Asian countries. As acupuncture can be effective for neurological conditions [3], applying acupuncture to tinnitus has been considered. Some studies have reported that electrical stimulation, including electroacupuncture (EA) to the periauricular region can reduce tinnitus [4–6]. EA sends weak electrical stimulation through acupuncture needles inserted in an acupoint, and the stimulation can be strengthened by the synergistic effect of acupuncture and electrical stimulation [7]. Wang et al. [5] reported that EA is more effective than manual acupuncture and sham acupuncture in decreasing frequency and loudness, and increasing quality of life, in patients with idiopathic tinnitus. However, the EA group in Wang’s study received both systemic manual acupuncture including periauricular acupoints and four limbs plus EA in periauricular acupoints. Therefore, it is uncertain which treatment was effective in that study because based on Traditional Chinese Medicine meridian system theory, acupoints for tinnitus are not only located in the periauricular lesion but also in the distal lesion and the four limbs [8].

The systemic manual acupuncture based on Traditional Chinese Medicine is very difficult, even delicate technique to learn for clinicians who didn’t familiar with the Traditional Chinese Medicine. If specific site electroacupuncture without systemic manual acupuncture showed similar or better effect on tinnitus, clinicians could easily use this treatment for tinnitus patients. Therefore, in this study, we separated the systemic manual acupuncture and EA to clarify the effect of each treatment. We selected acupoints, according to meridian system theory. Then the manual acupuncture group (MA) received systemic manual acupuncture including periauricular and four limbs acupoints and the EA group only received acupuncture in specific site acupoints to receive electrical stimulation. In addition, we divided the EA group into two groups by treatment regions; periauricular EA group (PE) according to selection of points close to the disease site theory and distal EA group (DE) according to selection of distant point far from the disease site theory.

The aim of this study was to evaluate and compare the effects of systemic manual acupuncture, periauricular EA and distal EA to treat patients with tinnitus.

## Methods

This was a randomized, paralleled, open-label exploratory study. This study was conducted in Kyung Hee Korean Medical Hospital. The subjects were recruited from May 2013 to April 2014 through advertisements in local newspapers, hospital websites, and bulletin boards. The inclusion criteria were: (1) age 20–75 years and (2) tinnitus for > 2 weeks. Subjects were excluded if they (1) had a disease causing tinnitus (Meniere’s disease, otitis media, head injury, cerebral vascular accident, etc.), (2) had received acupuncture to treat tinnitus during the last 3 months, (3) were pregnant or breast-feeding, (4) had a cardiac disorder, or (5) were taking psychoactive drugs for tinnitus.

Oral and written informed consent was obtained from all eligible subjects, and they were assigned randomly to the systemic manual acupuncture group (MA), periauricular electroacupuncture group (PE), and distal electroacupuncture group (DE). A random drawing of straws by the subjects themselves was used with 12 equally distributed lots. The randomization results were open to the subjects, but hidden from the assessor, except for the EA practitioner. The researcher who conducted the randomization had no contact with the assessor.

This study protocol was approved by the institutional review board of Kyung Hee Korean Medical Center (KOMCIRB-2012-03).

### Intervention/Procedure

The sample size was based upon the primary outcome measure of THI score. An estimation of the effect sizes is necessary because of a lack of comparable studies to compare the effect of manual acupuncture, periauricular EA and distal EA treatment of idiopathic tinnitus. On the basis of a previous similar study [5] and an expert opinion, we postulated a small effect size with d = 0.54 in the reduction in THI score between groups for this exploratory study. We recruited and enrolled total 45 participants for all three study arms. This sample size allowed us to detect an effect size of d = 0.54 based on a power (1 − β) of 80%, an α error of 0.05 and an estimated 12% dropout rate in one-way ANOVA test, calculated using G*Power software 3.1.

We used sterile acupuncture (Dong Bang Acupuncture Inc., Chungcheongnam-do, South Korea, 40 mm in length and 0.25 mm in diameter) and a digital low-frequency electrical stimulator (STN-111®; Stratek, Gyeonggi-do, South Korea). All treatments were performed by a licensed Korean medical doctor with more than 3 years of clinical acupuncture experience. Treatment side was selected based on the tinnitus side. If a subject experienced tinnitus on both sides, the treatment was performed on both sides.

**MA:** Nine acupoints were selected according to the meridian system theory; five in the periauricular region (TE17, TE21, SI19, GB2, and GB8), two in the upper limbs (TE3 and TE9), and two in the lower limbs (ST36 and ST37). The acupuncture needles were inserted until the participant felt “De-qi” and remained for 20 min.

**PE:** Two periauricular acupoints (TE17 and TE21) were selected. The acupuncture needles were inserted until the participant felt “De-qi”, and the needles were connected to a digital low-frequency electrical stimulator (mixed frequency 4/100 Hz interval 3 sec) for 20 min.

**DE:** Four acupoints were selected. The two in the upper limbs (TE3 and TE9), known to be effective in treating tinnitus, and two in the lower limbs (ST36 and ST37) that were known to control the autonomic nervous system and modulate all body energy. The acupuncture needles were inserted until the participant felt “De-qi”, and the needles were connected to a digital low-frequency electrical stimulator (mixed frequency 4/100 Hz interval 3 sec) for 20 min.

Each acupoint was selected according to a previous study [9] and Traditional Chinese Medicine meridian system theory, 2 periauricular acupoints were selected according to selection of points close to the disease site theory, and 4 acupoints in upper & lower limbs were selected according to selection of distant point far from the disease site theory [10]. We considered the size of ear area, and then we chose 2 periauricular acupionts located front and back of ear.

**Fig. 1:**
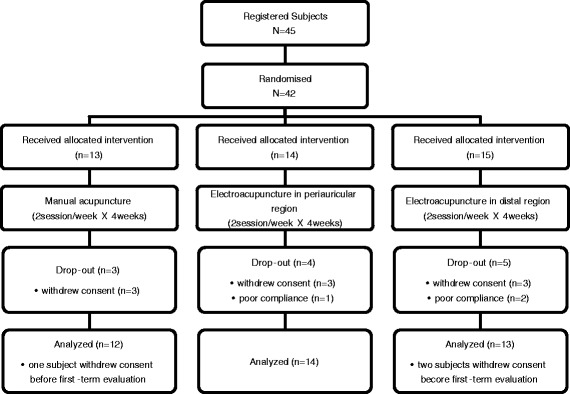
Study flow chart

All subjects received treatments twice weekly for a total of eight sessions over 4 weeks. We assessed compliance according to attendance at each session. If a subject did not receive treatment for 7 days or did not finish the eight sessions within 6 weeks, we dropped the subject because of poor compliance (Fig. 1).

### Assessment

The outcomes were the Korean version of the Tinnitus Handicap Inventory (THI) score, which has confirmed reliability and validity [11] and the visual analogue scale (VAS) scores for loudness (VAS _loudness_) and uncomfortable scores (VAS _uncomfortable_) [12, 13]. The THI score does not distinguish direction, so the THI score, we measured per person and the VAS was measured per ear. If a subject had bilateral tinnitus, the VAS was measured differently for each side.

The THI is a 25-item self-administered questionnaire that measures tinnitus severity. Each item receives a score of 0, 2, or 4 points, resulting in a total score of 100 (0 point = “no”, 2 points = “sometimes”, 4 points = “yes”). The VAS range was 0–100 (0 = no tinnitus and totally comfortable, 100 = maximum tinnitus and the worst tinnitus experienced). We drew a 100 mm line on paper and asked the subjects to mark a dot on the line. Then, we measured the length from point 0 to the dot with a ruler. Pure tone average (PTA) and speech discrimination (SD) were measured with the GSI 61 (Grason-Stadler, Inc., Eden Prairie, MN, USA). PTA was evaluated according to the mean of three frequencies (0.5, 1, and 2 KHz).

All assessments were measured four times: baseline (initial evaluation), mid-treatment (mid-term evaluation), at the end of treatment (end-term evaluation) and at the 4 weeks later the end of treatment (follow-up evaluation). The mid-term evaluation was measured just before the fifth treatment session.

### Statistical analysis

The statistical analysis was conducted on an intension-to-treat basis and 95% confidence intervals were calculated using SPSS ver. 17.0 for Windows (SPSS Inc., Chicago, IL, USA). Missing values from participants who dropped out were input based on the last observation carried forward method because tinnitus is a chronic and constant symptom. Demographic and clinical characteristics of the participants were compared between groups upon admission using one-way analysis of variance (ANOVA). One-way ANOVA was also used to examine the THI, VAS _loud_, and VAS _uncomfortable_ scores. The least significant difference was used as the post-hoc test. All adverse events were reported on the case report forms. A *p*-value < 0.05 was considered significant.

## Results

Of the 45 subjects enrolled, 42 were assigned randomly to the MA (*n* = 13), PE (*n* = 14), and DE (*n* = 15). Thirty subjects completed the protocol and 12 dropped out; nine withdrew consent and three were in poor compliance. As three subjects withdrew consent before the first-term evaluation, we analyzed measurements taken from 39 subjects. No differences in sex, exposure to noise experience, sleeping disorder, affected ear, age, tinnitus duration, PTA, or SD were detected between the groups (Table 1).

**Table 1:** Patient demographics

Characteristics	MA (*n* = 12)	PE (*n* = 14)	DE (*n* = 13)	*p*-value
Sex (male: female)	6:6	12:2	10:3	0.132
Exposure to noise	2 (16.6%)	1 (7.1%)	3 (23%)	0.561
Sleeping disorder	2 (16.6%)	3 (21.4%)	5 (38.5%)	0.431
Tinnitus side (R:L:B)	1:2:9	3:3:8	2:2:9	0.422
Age (y)	49.33 ± 15.57	54.57 ± 14.27	53.92 ± 13.21	0.613
Duration (y)	7.13 ± 7.85	9.56 ± 11.55	5.67 ± 6.40	0.529
PTA	27.80 ± 23.08	24.77 ± 21.75	16.31 ± 11.94	0.141
SD	84.00 ± 28.98	88.91 ± 12.82	91.27 ± 7.57	0.432

*MA* systemic manual acupuncture group, *PE* periauricular electroacupuncture group, *DE* distal electroacupuncture group, *PTA* pure tone average, *SD* speech discrimination. Discrete variables are expressed numbers (%) and analyzed with the chi-square test. Continuous variables are expressed as mean ± standard deviation and analyzed by one-way analysis of variance. *P*-values < 0.05 were considered significant

In between groups, there was no statistically significant difference in THI and VAS _loudness_ scores at any time point (Table 2), (Table 3).

**Table 2:** Changes in the THI scores

	Initial	Mid	End	Follow-up
MA (*n* = 12)	51.33 ± 24.80	45.00 ± 25.26	41.83 ± 32.46	40.00 ± 33.66
PE (*n* = 14)	55.14 ± 24.39	50.86 ± 22.64	50.71 ± 26.93	50.00 ± 27.38
DE (*n* = 13)	35.38 ± 16.03	35.38 ± 17.02	34.00 ± 18.17	33.85 ± 18.14
*p*-value	0.064	0.282	0.138	0.056

*MA* systemic manual acupuncture group, *PE* periauricular electroacupuncture group, *DE* distal electroacupuncture group, *THI* Tinnitus Handicap Inventory. All variables are expressed as mean ± standard deviation analyzed by one-way analysis of covariance considering THI score at baseline as covariate. P-values < 0.05 were considered significant

**Table 3:** Changes in the VAS _loudness_ and VAS _uncomfortable_ scores

	Initial	Mid	End	Follow-up
VAS _loudness_				
MA (*n* = 21)	48.76 ± 22.06	46.38 ± 22.16	42.62 ± 25.26	43.38 ± 24.61
PE (*n* = 22)	54.45 ± 23.75	55.09 ± 21.33	53.59 ± 24.39	49.68 ± 23.09
DE (*n* = 22)	47.73 ± 20.32	45.55 ± 20.81	45.05 ± 22.72	45.00 ± 21.48
p-value	0.787	0.548	0.374	0.496
VAS _uncomfortable_				
MA (*n* = 21)	42.14 ± 29.87	40.95 ± 30.51	38.67 ± 29.36^a^	36.67 ± 29.80^a^
PE (*n* = 22)	59.14 ± 26.12	59.41 ± 22.11	58.68 ± 23.30^b^	57.18 ± 24.56^b^
DE (*n* = 22)	42.55 ± 25.78	40.27 ± 27.02	37.82 ± 24.43^a^	42.73 ± 22.88^a^
p-value	0.099	0.067	0.011*	0.028*

*MA* systemic manual acupuncture group, *PE* periauricular electroacupuncture group, *DE* distal electroacupuncture group, *VAS* visual analogue scale. All variables are expressed as mean ± standard deviation analyzed by one-way analysis of covariance considering THI score at baseline as covariate. A *p*-value < 0.05 was considered significant. Different letter superscripts in the same column indicate a significant difference (*p < 0.05, LSD post-hoc test)

However, the VAS _uncomfortable_ scores were significantly different between the groups at the end-term (*p* = 0.011), and follow-up (*p* = 0.028) evaluations but not at the initial (*p* = 0.099) and mid-term (*p* = 0.067) evaluation. The MA and DE scores decreased significantly compared with those of the PE (Table 3).

**Fig. 2:**
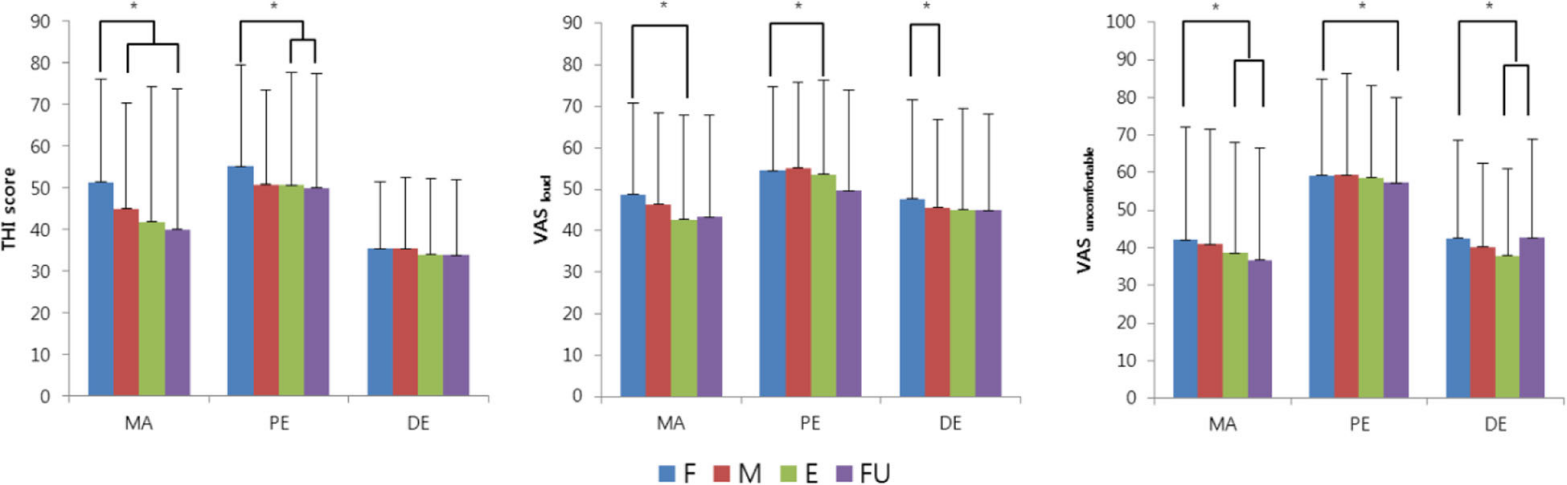
Changes in the THI, VAS _loudness_, and VAS _uncomfortable_ scores within groups

In within group, a significant decrease in the THI scores was detected in MA at the mid-term and the follow-up evaluations and a significant decrease in the THI scores was detected in the PE at the end-term and follow-up evaluations. In DE, there was no statistically significant difference at any time point. Both VAS _loud_ and VAS _uncomfortable_ scores decreased significantly within the group over time. The VAS _loudness_ scores in the MA at the end-term evaluation, the PE at the follow-up evaluation, and the DE at the mid-term evaluation decreased significantly compared to those of the other groups at the same time points. The VAS _uncomfortable_ scores in the MA and DE decreased significantly at the end-term and follow-up evaluations. The VAS _uncomfortable_ scores of the PE were significantly different at the follow-up evaluation (Fig. 2)

### Compliance and safety

Two subjects in the DE and one in the PE were dropped from the study because they did not attend the clinic appointments.

Two subjects in the PE complained of temporary pressure/pain in the periauricular region but these symptoms disappeared once the treatment ended. No other adverse effects were observed.

## Discussion

**Fig. 3:**
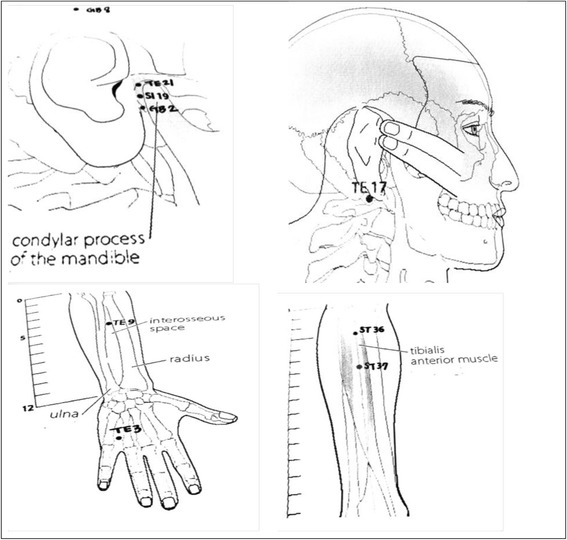
Selected acupoints and their positions

The acupoints of this trial were chosen based on a previous study [9] and Traditional Chinese Medicine meridian system theory [10]. We chose five acupoints in the periauricular region (TE17, TE21, SI19, GB2, GB8) for the MA that have been used frequently to treat ear diseases, two acupoints in the upper limbs (TE3 and TE9) that are effective for treating ear diseases, and two acupoints in the lower limbs (ST36 and ST37) that control the autonomic nervous system and modulate whole body energy [10]. We chose TE17 in the mastoid process area and TE 21 in front of the ear area for the PE to maximize the effect of EA on tinnitus [10]. We chose TE3 and TE9 for tinnitus and ST 36 and ST 37 for balance between the body and mind for the DE [14–16]. The selected acupoints and their positions are shown in Fig. 3.

It was difficult to include EA in this study because there is no standard method to set up a control group for EA. Generally, a non-acupoint electrostimulated control group, [17, 18] an acupoint with no electrical stimulation control group [5], or a combination of no acupoint and no electrical stimulation [19] are used as a control group. However, applying the former method is almost impossible in a small periauricular region such as the ear because it is difficult to choose a non-acupoint that does not affect electrical stimulation at the actual acupoint. It is difficult to resolve the effect of EA for the latter method because MA can have some therapeutic effect itself. Therefore, we compared the three groups without a control group.

Several studies have reported various effects of EA, and EA has been used to effectively treat migraine [20], obesity [21], cerebral infarction [22, 23], and tinnitus [5]. Most of these studies used EA combined with systemic manual acupuncture [5, 20, 21]; however, according to the Traditional Chinese Medicine meridian system theory, the body is an organic structure connected by meridians, meaning that several acupoints have a similar treatment effect not only in the target area but also in the upper and lower limbs. Therefore, we did not combine EA with systemic manual acupuncture to clarify the individual effects of and EA and systemic manual acupuncture.

In this study, there was no statistically significant difference in THI and VAS _loudness_ between groups. Only in VAS _uncomfortable,_ MA and DE showed **a** better effect than PE. However, VAS _uncomfortable_ means not the tinnitus itself but the discomfort feeling about the tinnitus. Therefore, it is hard to say that MA and DE have better effect than PE in tinnitus.

Within the group, MA and PE showed some significant effects on tinnitus based on the THI, VAS _loudness_, and VAS _uncomfortable_ scores, but DE only showed the VAS _uncomfortable_ score changed over time.

Considering that there was no change in THI and VAS _loudness_ at DE in within the group, it was an interesting result that in between groups the VAS _uncomfortable_ scores decreased significantly in MA and DE. If we consider that DE did not receive acupuncture or electrical stimulation in the periauricular region, these results could be explained as a systemic acupuncture effect of the meridian system, such as modulation of the autonomic nervous system and balancing of body and mind [14–16]. Therefore, we hypothesized that in the meridian system theory the meaning of acupoints distant away from target site would be the modulator of balancing.

We supposed that the effect of EA on tinnitus could be due to the synergism of combining systemic manual acupuncture with periauricular EA based on the present results and those of a previous study [5] However, further study is needed to confirm this supposition.

This study has several limitations. The first, in this study, we only evaluated the effect of EA and systemic manual acupuncture on tinnitus, therefore there was no control group in this study. To make sure the effect of EA or/and systemic manual acupuncture on tinnitus, next study that includes control group will be necessary. The second, the number of subjects in this study is relatively small. The third, this study was conducted in Korea, therefore many subjects were familiar with acupuncture treatment and it is possible that they already had prejudices or expectations about this study. Therefore, we suggest that randomized, placebo-controlled and large scaled study about EA and systemic manual acupuncture should be conducted in any country unfamiliar with acupuncture treatment.

## Conclusions

There was no statistically significant difference between systemic manual acupuncture, periauricular electroacupuncture and distal electroacupuncture in tinnitus. However, all three treatments had some effects on tinnitus in within the group before and after treatment and systemic manual acupuncture and distal electroacupuncture had some effects on VAS _uncomfortable_

## Abbreviations

DE: Distal electroacupuncture group
EA: Electroacupuncture
MA: Systemic manual acupuncture group
PE: Periauricular electroacupuncture group
PTA: Pure tone average
SD: Speech discrimination
THI: Tinnitus handicap inventory
VAS loudness: Visual analogue scale scores for loudness
VAS uncomfortable: Visual analogue scale scores for uncomfortable
VAS: Visual analogue scale.

## References

[1] Heller AJ (2003). Classification and epidemiology of tinnitus. Otolaryngol Clin North Am.

[2] Shargorodsky J, Curhan GC, Farwell WR (2010). Prevalence and characteristics of tinnitus among US adults. Am J Med.

[3] Lee H, Park HJ, Park J, Kim MJ, Hong M, Yang J (2007). Acupuncture application for neurological disorders. Neurol Res.

[4] Steenerson RL, Cronin GW (1999). Treatment of tinnitus with electrical stimulation. Otolaryngol Head Neck Surg.

[5] Wang K, Bugge J, Bugge S (2010). A randomised, placebo-controlled trial of manual and electrical acupuncture for the treatment of tinnitus. Complement Ther Med.

[6] Lee SK, Chung H, Chung JH, Yeo SG, Park MS, Byun JY (2014). Effectiveness of transcutaneous electrical stimulation for chronic tinnitus. Acta Otolaryngol..

[7] He M, Li X, Liu Y, Zhong J, Jiang L, Liu Y (2016). Electroacupuncture for tinnitus: A systemic review. PLoS One.

[8] Kim JI, Choi JY, Lee DH, Choi TY, Lee MS, Ernst E (2012). Acupuncture for the treatment of tinnitus: a systematic review of randomized clinical trials. BMC Complement Altern Med..

[9] Jeon SW, Kim KS, Nam HJ (2012). Long-term effect of acupuncture for treatment of tinnitus: a randomized, patient- and assessor-blind, sham-acupuncture-controlled, pilot trial. J Altern Complement Med.

[10] Association of Korean Medicine Colleges, Institute of Meridian and Acupoints: Details of Meridians & Acupoints Vol.1,2; A Guidebook for College Students. Seoul: Yuibang Publisher. p. 287-289, p. 508-510, p. 872-874, p. 893-894, p. 912-915, p. 922-925, p. 940-942, p. 955-958.

[11] Kim JH, Lee SY, Kim CH, Lim SL, Shin JN, Chung WH (2002). Reliability and validity of a Korean adaptation of the Tinnitus Handicap Inventory. Korean J Otolaryngol-Head Neck Surg..

[12] Park JH, Noh TS, Lee JH, Oh SH, Kim JS, Chung CK (2015). Difference in Tinnitus Treatment Outcome According to the Pulse Number of Repetitive Transcranial Magnetic Stimulation. Otol Neurotol.

[13] Li TT, Wang ZJ, Yang SB, Zhu JH, Zhang SZ, Cai SJ (2015). Transcutaneous electrical stimulation at auricular acupoints innervated by auricular branch of vagus nerve pairing tone for tinnitus: study protocol for a randomized controlled clinical trial. Trials..

[14] Iwa M, Matsushima M, Nakade Y, Pappas TN, Fujimiya M, Takahashi T (2006). Electroacupuncture at ST-36 accelerates colonic motility and transit in freely moving conscious rats. Am J Physiol Gastrointest Liver Physiol.

[15] Yin L, Jin X, Qiao W, Sun J, Shi X, Tian J (2003). PET imaging of brain function while puncturing the acupoint ST36. Chin Med J.

[16] Liu Y, Chen YL (2010). Analysis of information detection of biological energy on Shangjuxu(ST37) with acupuncure. Chin Acupunct Moxibustion.

[17] Zhou S, Haung LP, Liu J, Yu JH, Tian Q, Cao LJ (2012). Bilateral effects of 6 weeks’ unilateral acupuncture and electroacupuncture on ankle dorsiflexors muscle strength: a pilot study. Arch Phys Med Rehabil.

[18] Penza P, Bricchi M, Scola A, Campanella A, Lauria G (2011). Electroacupuncture is not effective in chronic painful neuropathies. Pain Med..

[19] Yeung WF, Chung KF, Tso KC, Zhang SP, Zhang ZJ, Ho LM (2011). Electroacupuncture for residual insomnia associated with major depressive disorder: a randomized controlled trial. Sleep.

[20] Zhen F, Bi-meng J (2014). Observation on Clinical Effects of Electroacupuncture for Migraine Without Aura. J Acupunct Tuina Sci.

[21] Darbandi S, Darbandi M, Mokarram P, Owji AA, Zhao B, Ghayor-Mobarhan M (2013). Effects of Body Electroacupuncture on Plasma Leptin Concentrations in Obese and Overweight People in Iran: A Randomized Controlled Trial. Altern Ther Health Med.

[22] Wang C, Wu Z, Li N, Zhao Y, Tian F, Zhou X (2014). Clinical curative effect of electric acupuncture on acute cerebral infarction: a randomized controlled multicenter trial. J Tradit Chin Med.

[23] Hsing WR, Imamura M, Weaver K, Fregni F, Azevedo Neto RS (2012). Clinical effects of scalp electrical acupuncture in stroke: a sham-controlled randomized clinical trial. J Altern Complement Med.

